# Identifying multispecies synchrony in response to environmental covariates

**DOI:** 10.1002/ece3.2518

**Published:** 2016-11-04

**Authors:** Ben Swallow, Ruth King, Stephen T. Buckland, Mike P. Toms

**Affiliations:** ^1^ Centre for Research into Ecological and Environmental ModellingSchool of Mathematics and StatisticsUniversity of St AndrewsSt AndrewsUK; ^2^ Atmospheric Chemistry Research GroupSchool of ChemistryUniversity of BristolBristolUK; ^3^ School of MathematicsUniversity of EdinburghEdinburghUK; ^4^ British Trust for OrnithologyThetfordNorfolkUK

**Keywords:** ecosystem modeling, multispecies, predation, synchrony, Tweedie

## Abstract

The importance of multispecies models for understanding complex ecological processes and interactions is beginning to be realized. Recent developments, such as those by Lahoz‐Monfort et al. (2011), have enabled synchrony in demographic parameters across multiple species to be explored. Species in a similar environment would be expected to be subject to similar exogenous factors, although their response to each of these factors may be quite different. The ability to group species together according to how they respond to a particular measured covariate may be of particular interest to ecologists. We fit a multispecies model to two sets of similar species of garden bird monitored under the British Trust for Ornithology's Garden Bird Feeding Survey. Posterior model probabilities were estimated using the reversible jump algorithm to compare posterior support for competing models with different species sharing different subsets of regression coefficients. There was frequently good agreement between species with small asynchronous random‐effect components and those with posterior support for models with shared regression coefficients; however, this was not always the case. When groups of species were less correlated, greater uncertainty was found in whether regression coefficients should be shared or not. The methods outlined in this study can test additional hypotheses about the similarities or synchrony across multiple species that share the same environment. Through the use of posterior model probabilities, estimated using the reversible jump algorithm, we can detect multispecies responses in relation to measured covariates across any combination of species and covariates under consideration. The method can account for synchrony across species in relation to measured covariates, as well as unexplained variation accounted for using random effects. For more flexible, multiparameter distributions, the support for species‐specific parameters can also be measured.

## 1 Introduction

When modeling the dynamics of ecological populations, most standard approaches have tended to consider species independently of each other by fitting a single model to each of the species (Harris, 2015; Lecomte, Benoït, Etienne, Bel, & Parent, 2013). Parameters in these models are then estimated and interpreted independently of each other. However, this approach oversimplifies the complex interactions that inevitably underpin the ecological dynamics present within such ecosystems. The ability to understand these ecological dynamics is often difficult in practice because traditional models typically estimate just a single set of demographic rates (e.g., survival or productivity, but not both). Linking demographic rates across numerous species, without merely measuring associations between species, adds an additional nontrivial level of complexity (Buonaccorsi, Elkinton, Evans, & Liebhold, 2001; Ovaskainen, Hottola, & Siitonen, 2010).

In order to better understand such dynamics at an ecosystem level, it is important to account for these multispecies interactions. Further extensions to standard single‐species models must be made if one is to account for more complex dependencies and correlation structures. Joint species distribution models (JSDMs), which pool data from multiple sources (Fithian, Elith, Hastie, & Keith, 2015) or from multiple species (Clark, Gelfand, Woodall, & Zhu, 2013; Pollock et al., 2014; Thorson et al., 2015), allow more parsimonious models to be fitted while also propagating all forms of uncertainty throughout the model. The development of such models outside of the JSDM literature has been slow; however, recent advances by Lahoz‐Monfort, Morgan, Harris, Wanless, and Freeman (2011) have enabled such models to be formulated. The authors, extending the work of Grosbois et al. (2009), proposed a statistical model in which random effects were used to estimate the level of synchrony across multiple species. Within the model, a synchronous component is used to represent the common response of all the species considered, while an asynchronous component accounts for any additional variation specific to each species. However, these components are conditional on environmental covariates in the model, which then only estimate synchrony in unexplained variation.

Lahoz‐Monfort et al. (2011) fitted the model with species‐specific coefficients for all covariates. Only modeling synchrony in variance unexplained by the covariates in the model risks underestimating the magnitude of the synchrony inherent in the modeled species. If the variation explained by any covariates in the model is largely synchronous across species, then corresponding species‐invariant random‐effect variances will consequently be reduced in relation to the species‐specific ones, and the amount of synchrony estimated across the species will be lower than in reality. Additionally, in this case, precision in parameter estimates will be lower if they could realistically be shared across multiple species.

To estimate the degree of synchrony with respect to the covariates, Lahoz‐Monfort et al. (2011) fitted two models, one with covariates and one without (the null model), and then compared the random‐effect variances in each case. Changes in the observed magnitude of the random‐effect variances were then used to indicate whether the additional unexplained variation was largely synchronous or asynchronous. If the species‐invariant random‐effect variance increases, then this suggests that the response to covariates is overall largely synchronous. Conversely, if the species‐specific variances increase, then it can be concluded that the response to covariates is largely asynchronous. However, in neither case can the synchronous aspect of the response to each individual covariate be easily analyzed for each species–covariate combination. Fitting every model with unique species–covariate combinations in order to compare the ratio of random‐effect variances would be completely infeasible. Apart from the obvious computational demand of this approach, which increases proportionally with each covariate added to the model, this approach also assumes that unexplained variation no longer attributed to a given covariate will be completely absorbed into either of the two random effects. In reality, it is highly likely that part or all of this variation could be attributed to either a fixed intercept or other covariates in the model.

We propose an alternative approach, estimating posterior model probabilities associated with different models, where each regression coefficient can be shared across subsets of the species considered. The method explores uncertainty across both parameter and model space; posterior support for models with regression coefficients shared across different subsets of the species under consideration can be estimated. Each covariate is allowed to be shared across different subsets of the species considered, such that species with “similar" parameter estimates associated with each covariate can be grouped together.

The covariate synchrony method is applied to long‐term longitudinal data relating to numbers of six species of birds visiting garden feeding stations across the UK. These six species are split into two ecologically similar groups, namely blue tit (BT) *Cyanistes caeruleus*, great tit (GT) *Parus major*, and coal tit (CT) *Periparus ater* in the first, and house sparrow (HS) *Passer domesticus*, greenfinch (GF) *Chloris chloris*, and chaffinch (CF) *Fringilla coelebs* in the second. Some of these species have shown severe declines over the past few decades, while others have remained stable or are increasing (Newson, Rexstad, Baillie, Buckland, & Aebischer, 2010). Various explanations have been put forward to explain the declines observed in some of these species, but there have been disagreements over what the main drivers are. This has been particularly apparent in relation to the possible role of predation. While previous studies have attempted to understand the changes in numbers of some small passerines (e.g. Bell, Baker, Parkes, Brooke, & Chamberlain, 2010; Chamberlain, Glue, & Toms, 2009; Newson et al., 2010; Swallow, Buckland, King, & Toms, 2015; Thomson, Green, Gregory, & Baillie, 1998), there has been little attempt to understand these populations at a multispecies level (although see Sullivan, Newson, & Pearce‐Higgins, 2015). As the species concerned share a similar environment and are susceptible to the same exogenous factors, it would be expected that some or all of these species may interact with each other or respond in a similar way to the environment around them.

In particular, we concentrate on spatial synchrony in species’ response to covariates using log‐linear models; however, the method is easily applicable to many other cases and to model frameworks where parameters or coefficients can be shared across species, locations, or time periods.

## 2 Materials and Methods

### 2.1 Data description

The data used come from the British Trust for Ornithology's (BTO) Garden Bird Feeding Survey (GBFS) and relate to an annual mean of up to 26 weekly maximum counts conducted between October and March each year at approximately 200 sites. Inevitably, given the long time period involved, there is a degree of site turnover; however, replacement sites are selected to match as closely as possible—in terms of location and garden type—the site being replaced. The data analyses in this study span the years 1970/71 to 2005/06 inclusive (henceforth the year 1970 signifies the winter of 1970/71) and correspond to 693 individually monitored sites spanning the UK. The spatial distribution of GBFS sites reflects that of the human population, such that there are more sites in areas with greater densities of people. Participants in the survey note the maximum number of each species they observe at any given time feeding at their garden feeding stations or, in the case of predators, hunting the birds visiting the feeders, in up to 26 weeks each winter season.

In particular, we chose two distinct sets of three species of potential sparrowhawk prey monitored under the GBFS that would be expected to have similar ecological requirements (Newton, 1986). The first is a group of closely related species of the same family, namely blue tit, great tit, and coal tit. The second are three species largely associated with a winter diet of medium‐ to large‐sized seeds, namely house sparrow, greenfinch, and chaffinch. An average over the weekly maxima was calculated for each site and year, giving an essentially continuous distribution.

**Figure 1:**
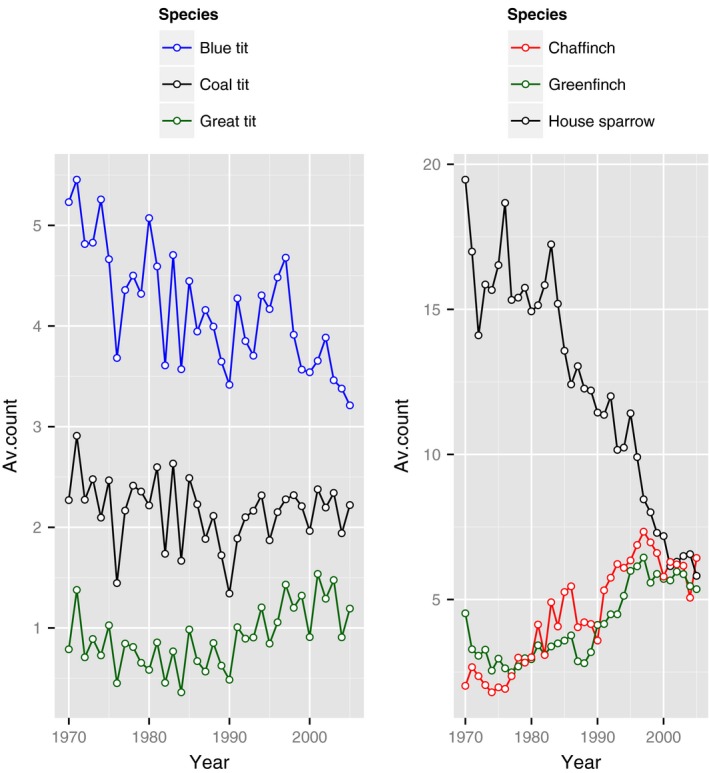
Average trends in the number of each of the three tit species (left) and grain‐feeding birds (right) observed across sites monitored by the GBFS from 1970 to 2005

Annual averages across all sites monitored under the GBFS surveys show similar peaks and troughs for each of the three tit species, as well as similarities for greenfinch and chaffinch (Figure 1). Marginal correlations across both sites and years between the observations of each pair of species in the two groups were calculated (Table 1). For the tit species, a significant positive correlation was found between all pairings when averages across years were used (.78 [BT vs. GT], .46 [BT vs. CT], and.60 [GT vs. CT]; Table 1a). This suggests that sites that can, on average, support or attract greater numbers of one of these species also attract greater numbers of the other species too. Correlation across years was also significant and positive for the blue tit–great tit and great tit–coal tit pairings, but not so for blue tit–coal tit. For the other three species, results were more variable. There was a strong positive correlation between the finch species across both space and time (.4 and .84, respectively), but less so for house sparrow (Table 1a), a species whose populations have been in long‐term decline within the study period, and for which there has been a change in the temporal pattern of peak garden use (Robinson, Siriwardena, & Crick, 2005). In fact, temporal correlation between house sparrow and each of the finch species was strongly negative in both (−.81 [HS vs. GF] and −.84 [HS vs. CF]). Populations of both finches are likely to be augmented over the winter with migrants from the continent. As house sparrows are largely sedentary, we would not expect the years where the finch populations are particularly large to correspond to high numbers of sparrows.

**Table 1:** Pairwise Pearson's correlations between site annual means for blue tit (BT), great tit (GT), and coal tit (CT) (left‐hand columns), and house sparrow (HS), greenfinch (GF), and chaffinch (CF) (right‐hand columns). (a) Marginal correlation between sites; that is, means are taken across years within sites and compared between species pairs. (b) Marginal correlation between years; that is, means are taken across sites for each year and compared between species pairs

Species pair	Correlation (*p*‐value)	Species pair	Correlation (*p*‐value)
(a)			
BT vs. GT	.78 (<.001)	HS vs. GF	.09 (.023)
BT vs. CT	.46 (<.001)	HS vs. CF	−.08 (.028)
GT vs. CT	.60 (<.001)	GF vs. CF	.40 (<.001)
(b)			
BT vs. GT	.57 (<.001)	HS vs. GF	−.81 (<.001)
BT vs. CT	.00 (.986)	HS vs. CF	−.84 (<.001)
GT vs. CT	.57 (<.001)	GF vs. CF	.84 (<.001)

In gardens across the UK, these species are all subject to similar exogenous factors and it may be that the different species are responding similarly or differently to these same factors. We would expect, due to the similarities in ecology of these three species in each group, that there would be some degree of synchrony across them in relation to their response to environmental covariates. However, the results of Swallow, Buckland, King, & Toms, unpublished data indicate that the species within each of the two groups studied here also respond differently in response to some of the covariates. As such, they offer ideal groups to analyze both synchronous and asynchronous aspects of their population dynamics.

### 2.2 The model

The model is an extension of that presented in Swallow et al. (2015), to incorporate the simultaneous modeling of more than one response species and the sharing of relevant parameters where possible. We extended the modeling framework of Grosbois et al. (2009) and Lahoz‐Monfort et al. (2011), which accounts for variation not explained by the fixed effects through two independent random effects. The method adds to previous work done on multispecies synchrony by Lahoz‐Monfort et al. (2013), who studied multispecies productivity and Schaub, von Hirschheydt, and Grüebler (2015), who studied multisite synchrony in demographic rates and populations.

Formally, let $y_{s,i,t}$ be the observed mean of weekly maxima of species *s* at site *i* in year *t*, $x_{i}$ spatially explicit covariates with associated parameter vector **β**{*s*}, and $v_{i,t}$ spatially explicit and time‐explicit covariates with associated parameter vector **γ**{*s*}. We denote $\theta \{s_{1},s_{2}\}$ to be the parameter θ shared over species $s_{1}$ and $s_{2}$. The covariates $x_{i}$ and $v_{i,t}$ could also be species‐specific, but in this application they are not. In addition, we tested for interactions within and between prey species by including a year‐lagged measure of each species as a covariate ${\tilde{y}}_{s,i,t\;-\;1}$. The associated coefficient $\nu_{j,k}$ corresponds to the effect of species *j* on species *k*. In the case where *j* = *k*, this parameter is equivalent to the concept of density dependence (Dennis & Taper, 1994). As the empirical distributions for each of the three species have a nonzero probability of exact zeros, while also being bounded below by zero, effectively continuous with discrete mass at zero and positively skewed, special consideration was given to the distributional form of the model. To account for each of these aspects of the data, we used the Tweedie distributions (here denoted Tw) (Jørgensen, 1987). Given a positive dispersion parameter ϕ and index parameter *p*∉(0,1), the Tweedie distributions are defined by the power mean–variance relationship $\text{Var}\left(y\right)=\phi \mu^{p}$. For values of *p* ∈ (1,2), the distributions are non‐negative‐continuous with a discrete probability mass at the origin. The model is then defined as follows:

(1) $$y_{s,i,t}∼\text{Tw}\left(\mu_{s,i,t},\phi \left\{s\right\},p\left\{s\right\}\right),$$

where

(2) $$\mu_{s,i,t}=\mu_{s,i,t-1}\exp\left\{\alpha_{s}+x_{i}^{⊤}\beta \left\{s\right\}+v_{i,t}^{⊤}\gamma \left\{s\right\}+\sum_{l=1}^{n_{s}}{\tilde{y}}_{l,i,t-1}^{⊤}\nu_{l,s}+\epsilon \left(i\right)+\delta_{s}\left(i\right)\right\},$$

(3) $$\epsilon \left(i\right)∼N\left(0,\sigma_{\epsilon }^{2}\right),$$

and

(4) $$\delta_{s}\left(i\right)∼N\left(0,\sigma_{s}^{2}\right).$$

The first random effect, ε(*i*), is a site‐specific random effect that is constant across species, accounting for synchronous variation that is common to all species. The second, $\delta_{s}\left(i\right)$, is a site‐specific random effect that is estimated separately for each species and accounts for additional variation that is asynchronous. The $\delta_{s}\left(i\right)$ were assumed to be independent of each other and of the ε(*i*).

Additional intra‐ and interspecific interactions between response species can also be added to the model where appropriate to create an even more flexible model that accounts for all levels of interactions between the species considered.

Following some simple algebraic manipulation and implementing the hierarchical centering reparameterization method (Browne, 2004; Browne, Steele, Golalizadeh, & Green, 2009), equation (2) can be rewritten as:

(5) $$\log\left(\frac{\mu_{s,i,t}}{\mu_{s,i,t-1}}\right)=v_{i,t}^{⊤}\gamma \left\{s\right\}+\sum_{l=1}^{n_{s}}\nu_{ls}{\tilde{y}}_{lit-1}^{⊤}+\epsilon \left(i\right)+\delta_{s}\left(i\right),$$

where

(6) $$\epsilon_{i}∼N\left(0,\sigma_{\epsilon }^{2}\right)$$

and

(7) $$\delta_{s}\left(i\right)∼N\left(\alpha_{s}+x_{i}^{⊤}\beta \left\{s\right\},\sigma_{s}^{2}\right).$$

That is, we modeled the difference in log abundance of species *s* as a function of environmental covariates and random effects. The model also requires the estimation of $\mu_{s,i,0}$, which is a site‐ and species‐specific offset corresponding to the expected value in the year prior to the survey commencing at each site. As in Swallow et al. (2015), we used a data augmentation approach to estimate these parameters. That is, they are treated as additional unknowns to be estimated from the rest of the data. This methodology allows both zero observations and the first observation at each site to contribute to estimating the remaining regression parameters. This method can also be used when covariate values are missing or for missing years of observations during the survey (although that was not necessary here). The data‐augmented $\mu_{i0}$ are also used as the density‐dependence covariate for the initial year of observations.

The analyses are conducted in a Bayesian framework using a Markov chain Monte Carlo (MCMC) approach to obtain inference on the model parameters of interest. A single‐update Metropolis–Hastings algorithm is used to update the parameters, with an adaptive tuning approach used for the proposal distributions to improve efficiency of the algorithm. More details can be found in Swallow et al. (2015).

Estimates of the proportion of variance for each species that is synchronous with the other species considered in the model can be calculated. That is the intraclass correlation coefficient (ICC) defined as:

(8) $${\text{ICC}}_{s}=\frac{\sigma_{\epsilon }^{2}}{\sigma_{\epsilon }^{2}+\sigma_{s}^{2}}.$$

Values close to 1 suggest largely synchronous unexplained variation, while values close to zero suggest mostly asynchronous unexplained variation. This measure of synchrony, however, does not take into consideration any variation explained by the covariates. This variation may be an important driver of the synchrony or asynchrony inherent in the species population dynamics, and therefore, being able to identify which species responds similarly or differently to any of the measured covariates considered should also be of interest.

### 2.3 Detecting synchrony to measured covariates

In order to group together species with similar responses to the environmental covariates presented in Swallow et al. (2015) (or similar mean–variance relationships in the case of the Tweedie parameters), we used the reversible jump algorithm (e.g. King, Morgan, Gimenez, & Brooks, 2010) to estimate posterior model probabilities associated with different species groupings for each of the covariate coefficients and the two Tweedie parameters ϕ and *p*. The particular reversible jump algorithm used is based on that described by King and Brooks (2002), who fit a model for detecting age dependency in mark–recapture parameters. We used a similar algorithm here to group together species with similar responses to measured covariates. The full algorithm is detailed in the supporting information. The algorithm essentially selects one of the parameters that can be shared across species at random and then proposes to move to a model where either an existing shared group is split into two distinct groups with different parameter values or two existing groups of species with distinct parameter values are merged into one group with a single shared parameter value.

To test for interactions within and between prey species, a covariate‐dependence approach was taken to model selection. We estimated the posterior model probabilities associated with the model where $\nu_{j,k}=0$ vs. $\nu_{j,k}\neq 0$. Further details can also be found in Swallow et al. (2015).

### 2.4 Prior distributions

Conducting the analysis in a Bayesian framework requires prior distributions to be specified on all model parameters. We used uninformative priors for parameter distributions (Table 2). Prior distributions for each species were assumed equal and specified independently of each other. Density dependence was formulated in such a way that it can only intuitively have a negative coefficient; hence, a half‐normal prior is specified for these parameters. All species–covariate combinations were assumed equal a priori; however, this could easily be relaxed to give zero mass to ecologically infeasible combinations.

**Table 2:** Prior distributions for the model parameters

Parameter	Prior distribution
$\alpha_{s}$	$N(0,10^{-2})$
$\beta_{j}\left\{s\right\}$	$N(0,10^{-2})$
$\gamma_{j}\left\{s\right\}$	$N(0,10^{-2})$
$\nu_{i,j}\left(i=j\right)$	$HN^{-}\left(0,10^{-2}\right)$
$\nu_{i,j}\left(i\neq j\right)$	$N(0,10^{-2})$
$\mu_{s,i,0}$	*U*[0,200]
ϕ{*s*}	*U*[0,5]
*p*{*s*}	*U*[1,2]
$\sigma_{\epsilon }^{2}$	$\Gamma^{-1}\left(10^{-3},10^{-3}\right)$
$\sigma_{s}^{2}$	$\Gamma^{-1}\left(10^{-3},10^{-3}\right)$

To aid with specifying the parameters of the proposal distributions for the parameter update step, we initially ran the full model without the reversible jump step for 50,000 iterations, of which the first 30,000 iterations were discarded as burn‐in. The posterior means and standard deviations for the density‐dependence and interspecific interactions coefficients were then used as the proposal distribution means and standard deviations for the corresponding parameters in the full analysis. Independent normal distributions with a zero mean and standard deviation $10^{-3}$ were used as the proposal distributions for the reversible jump step. Good mixing between models appeared to be achieved when using these proposals. The full model defined above including model uncertainty was then run independently for 100,000 iterations with the first 50,000 iterations discarded as burn‐in for the two sets of species discussed above. Convergence was checked using visual observation of trace plots, which gave no evidence to suggest a lack of convergence.

## 3 Results

Marginal posterior means and 95% credible intervals for the model parameters and intraclass correlation coefficients, together with their marginal posterior model probabilities, are given in Tables 3, 4, 5, 6, 7 (tits) and 8, 9, 10, 11, 12 (ground‐feeding species).

**Table 3:** Blue tit, great tit, and coal tit multispecies model. Intercept and density‐dependence parameters are species‐specific, with the reversible jump algorithm used to test for synchrony across the three species for all other regression covariate parameters. Posterior means and 95% symmetric credible intervals are presented. Covariate dependence is also conducted on the density‐dependence parameters

Parameter	Covariate	Posterior mean	95% CI
$\alpha_{\mathrm{bt}}$	Intercept	−0.0352	(−0.0414, −0.0284)
$\alpha_{\mathrm{gt}}$	Intercept	−0.0269	(−0.0326, −0.0202)
$\alpha_{\mathrm{ct}}$	Intercept	−0.0477	(−0.0575, −0.0378)
$\beta_{1}\left\{\text{bt}\right\}$	Northing	−0.0102	(−0.0177, −0.0039)
$\beta_{1}\left\{\text{gt}\right\}$	Northing	−0.0102	(−0.0176, −0.0039)
$\beta_{1}\left\{\text{ct}\right\}$	Northing	0.0076	(−0.0026, 0.0180)
$\beta_{2}\left\{\text{bt}\right\}$	Easting	−0.0080	(−0.0143, −0.0009)
$\beta_{2}\left\{\text{gt}\right\}$	Easting	−0.0080	(−0.0142, −0.0009)
$\beta_{2}\left\{\text{ct}\right\}$	Easting	−0.0275	(−0.0375, −0.0172)
$\beta_{3}\left\{\text{bt}\right\}$	Sub/rur	−0.0155	(−0.0209, −0.0101)
$\beta_{3}\left\{\text{gt}\right\}$	Sub/rur	−0.0169	(−0.0228, −0.0114)
$\beta_{3}\left\{\text{ct}\right\}$	Sub/rur	−0.0133	(−0.0200, −0.0038)
$\nu_{\mathrm{bt},\mathrm{bt}}$	Dens dep	−0.0260	(−0.0298, −0.0221)
$\nu_{\mathrm{gt},\mathrm{gt}}$	Dens dep	−0.0298	(−0.0339, −0.0260)
$\nu_{\mathrm{ct},\mathrm{ct}}$	Dens dep	−0.0333	(−0.0404, −0.0464)
$\gamma_{1}\left\{\text{bt}\right\}$	Sparrowhawk	−0.0032	(−0.0073, 0.0010)
$\gamma_{1}\left\{\text{gt}\right\}$	Sparrowhawk	−0.0030	(−0.0070, 0.0012)
$\gamma_{1}\left\{\text{ct}\right\}$	Sparrowhawk	0.0170	(0.0088, 0.0255)
$\gamma_{2}\left\{\text{bt}\right\}$	Collared dove	−0.0005	(−0.0042, 0.0033)
$\gamma_{2}\left\{\text{gt}\right\}$	Collared dove	−0.0004	(−0.0042, 0.0034)
$\gamma_{2}\left\{\text{ct}\right\}$	Collared dove	0.0160	(0.0082, 0.0238)
$\gamma_{3}\left\{\text{bt}\right\}$	Ground frost	0.0185	(0.0133, 0.0238)
$\gamma_{3}\left\{\text{gt}\right\}$	Ground frost	0.0126	(0.0074, 0.0183)
$\gamma_{3}\left\{\text{ct}\right\}$	Ground frost	0.0134	(0.0073, 0.0197)
ϕ{bt}	—	0.1654	(0.1567, 0.1749)
ϕ{gt}	—	0.1985	(0.1912, 0.2060)
ϕ{ct}	—	0.3439	(0.3298, 0.3584)
*p*{bt}	—	1.4469	(1.4106, 1.4814)
*p*{gt}	—	1.1797	(1.1656, 1.1938)
*p*{ct}	—	1.2714	(1.2595, 1.2831)

**Table 4:** Blue tit, great tit, and coal tit multispecies model. Intercept and density‐dependence parameters are species‐specific, with the reversible jump algorithm used to test for synchrony across the three species for all other regression covariate parameters. Posterior means and 95% symmetric credible intervals are presented. Covariate dependence is also conducted on the density‐dependence parameters

Parameter	Posterior mean	95% CI
$\sigma_{\epsilon }^{2}$	0.0043	(0.0036, 0.0051)
$\sigma_{\mathrm{bt}}^{2}$	0.0005	(0.0003, 0.0007)
$\sigma_{\mathrm{gt}}^{2}$	0.0004	(0.0002, 0.0007)
$\sigma_{\mathrm{ct}}^{2}$	0.0049	(0.0032, 0.0059)
ICC${}_{\mathrm{bt}}$	0.903	(0.852, 0.947)
ICC${}_{\mathrm{gt}}$	0.912	(0.857, 0.957)
ICC${}_{\mathrm{ct}}$	0.494	(0.411, 0.591)

**Table 5:** $\beta_{s}$ marginal posterior probabilities (MPP) testing for synchrony in response to environmental covariates from the model in Table 3, corresponding to northing, easting, and suburban/rural, respectively. {bt, gt} denotes the parameter shared across blue tit and great tit

Northing		Easting		Sub/rur	
Model	MPP	Model	MPP	Model	MPP
{bt, gt}, {ct}	.931	{bt, gt}, {ct}	.979	{bt, ct}, {gt}	.892
{bt, ct}, {gt}	.041	{bt}, {gt}, {ct}	.021	{gt, ct}, {bt}	.107
{bt}, {gt}, {ct}	.028			{bt}, {gt}, {ct}	.001

**Table 6:** $\gamma_{s}$ marginal posterior probabilities (MPP) testing for synchrony in response to environmental covariates from the model in Table 3, corresponding to sparrowhawk collared dove and ground frost, respectively. {bt, gt} denotes the parameter shared across blue tit and great tit

Sparrowhawk		Collared dove		Ground frost	
Model	MPP	Model	MPP	Model	MPP
{bt, gt}, {ct}	.968	{bt, gt}, {ct}	.997	{gt, ct}, {bt}	.994
{bt}, {gt}, {ct}	.032	{bt}, {gt}, {ct}	.003	{bt, ct}, {gt}	.006

**Table 7:** Marginal posterior probabilities (MPP) relating to the sharing of the two Tweedie variance parameters across tit species from the model in Table 3. {bt, gt} denotes the parameter shared across blue tit and great tit

ϕ		*p*	
Model	MPP	Model	MPP
{bt}, {gt}, {ct}	1.000	{bt}, {gt}, {ct}	1.000

**Table 8:** House sparrow, greenfinch, and chaffinch multispecies model. Intercept and density‐dependence parameters are species‐specific, with the reversible jump algorithm used to test for synchrony across the three species for all other regression covariate parameters. Posterior means and 95% symmetric credible intervals are presented. Covariate dependence is also conducted on the density‐dependence parameters

Parameter	Covariate	Posterior mean	95% CI
$\alpha_{\mathrm{hs}}$	Intercept	−0.0600	(−0.0720, −0.0485)
$\alpha_{\mathrm{gf}}$	Intercept	−0.0353	(−0.0453, −0.0250)
$\alpha_{\mathrm{cf}}$	Intercept	−0.0058	(−0.0138, 0.0021)
$\beta_{1}\left\{\text{hs}\right\}$	Northing	−0.0060	(−0.0192, 0.0048)
$\beta_{1}\left\{\text{gf}\right\}$	Northing	−0.0012	(−0.0102, 0.0076)
$\beta_{1}\left\{\text{cf}\right\}$	Northing	−0.0008	(−0.0084, 0.0072)
$\beta_{2}\left\{\text{hs}\right\}$	Easting	−0.0285	(−0.0400, −0.0178)
$\beta_{2}\left\{\text{gf}\right\}$	Easting	−0.0246	(−0.0360, −0.0146)
$\beta_{2}\left\{\text{cf}\right\}$	Easting	−0.0226	(−0.0315, −0.0138)
$\beta_{3}\left\{\text{hs}\right\}$	Sub/rur	−0.0156	(−0.0252, −0.0043)
$\beta_{3}\left\{\text{gf}\right\}$	Sub/rur	−0.0181	(−0.0266, −0.0087)
$\beta_{3}\left\{\text{cf}\right\}$	Sub/rur	−0.0202	(−0.0277, −0.0129)
$\nu_{\mathrm{hs},\mathrm{hs}}$	Dens dep	NA	NA
$\nu_{\mathrm{gf},\mathrm{gf}}$	Dens dep	−0.0142	(−0.0195, −0.0093)
$\nu_{\mathrm{cf},\mathrm{cf}}$	Dens dep	−0.0282	(−0.0330, −0.0228)
$\nu_{\mathrm{gf},\mathrm{hs}}$	Interaction	0.0327	(0.0258, 0.0401)
$\nu_{\mathrm{cf},\mathrm{hs}}$	Interaction	−0.0462	(−0.0555, −0.0367)
$\gamma_{1}\left\{\text{hs}\right\}$	Sparrowhawk	−0.0459	(−0.0532, −0.0387)
$\gamma_{1}\left\{\text{gf}\right\}$	Sparrowhawk	−0.0016	(−0.0064, 0.0035)
$\gamma_{1}\left\{\text{cf}\right\}$	Sparrowhawk	−0.0016	(−0.0063, 0.0036)
$\gamma_{2}\left\{\text{hs}\right\}$	Collared dove	0.0037	(0.0003, 0.0071)
$\gamma_{2}\left\{\text{gf}\right\}$	Collared dove	0.0036	(0.0001, 0.0071)
$\gamma_{2}\left\{\text{cf}\right\}$	Collared dove	0.0118	(0.0070, 0.0175)
$\gamma_{3}\left\{\text{hs}\right\}$	Ground frost	0.0375	(0.0321, 0.0431)
$\gamma_{3}\left\{\text{gf}\right\}$	Ground frost	0.0145	(0.0046, 0.0238)
$\gamma_{3}\left\{\text{cf}\right\}$	Ground frost	0.0375	(0.0321, 0.0431)
ϕ{hs}	—	0.6867	(0.6619, 0.7121)
ϕ{gf}	—	0.5360	(0.5182, 0.5545)
ϕ{cf}	—	0.3890	(0.3754, 0.4029)
*p*{hs}	—	1.3534	(1.3407, 1.3639)
*p*{gf}	—	1.4218	(1.4069, 1.4370)
*p*{cf}	—	1.3563	(1.3450, 1.3731)

**Table 9:** House sparrow, greenfinch, and chaffinch multispecies model. Intercept and density‐dependence parameters are species‐specific, with the reversible jump algorithm used to test for synchrony across the three species for all other regression covariate parameters. Posterior means and 95% symmetric credible intervals are presented. Covariate dependence is also conducted on the density‐dependence parameters

Parameter	Posterior mean	95% CI
$\sigma_{\epsilon }^{2}$	0.0037	(0.0027, 0.0050)
$\sigma_{\mathrm{hs}}^{2}$	0.0145	(0.0120, 0.0173)
$\sigma_{\mathrm{gf}}^{2}$	0.0084	(0.0068, 0.0103)
$\sigma_{\mathrm{cf}}^{2}$	0.0039	(0.0025, 0.0055)
ICC${}_{\mathrm{hs}}$	0.205	(0.154, 0.256)
ICC${}_{\mathrm{gf}}$	0.308	(0.221, 0.398)
ICC${}_{\mathrm{cf}}$	0.495	(0.344, 0.648)

**Table 10:** $\beta_{s}$ marginal posterior probabilities (MPP) testing for synchrony in response to environmental covariates from the model in Table 8, corresponding to northing, easting, and suburban/rural, respectively. {hs, gf} denotes the parameter shared across house sparrow and greenfinch

Northing		Easting		Sub/rur	
Model	MPP	Model	MPP	Model	MPP
{gf, cf}, {hs}	.591	{gf, cf}, {hs}	.649	{gf, cf}, {hs}	.494
{hs, cf}, {gf}	.223	{hs, gf}, {cf}	.238	{hs, cf}, {gf}	.252
{hs, gf}, {cf}	.175	{hs, cf}, {gf}	.105	{hs, gf}, {cf}	.250
{hs}, {gf}, {cf}	.010	{hs}, {gf}, {cf}	.008	{hs}, {gf}, {cf}	.003

**Table 11:** $\gamma_{s}$ marginal posterior probabilities (MPP) testing for synchrony in response to environmental covariates from the model in Table 8, corresponding to sparrowhawk collared dove and ground frost, respectively. {hs, gf} denotes the parameter shared across house sparrow and greenfinch

Sparrowhawk		Collared dove		Ground frost	
Model	MPP	Model	MPP	Model	MPP
{gf, cf}, {hs}	.991	{hs, gf}, {cf}	.981	{hs, cf}, {gf}	.994
{hs}, {gf}, {cf}	.009	{hs}, {gf}, {cf}	.019	{hs}, {gf}, {cf}	.006

**Table 12:** Marginal posterior probabilities (MPP) relating to the sharing of the two Tweedie variance parameters across tit species from the model in Table 8. {hs, gt} denotes the parameter shared across blue tit and great tit

ϕ		*p*	
Model	MPP	Model	MPP
{hs}, {gf}, {cf}	1.000	{hs, cf}, {gf}	.835
		{hs}, {gf}, {cf}	.165

Only synchrony across a maximum of two species was found for any covariate in either of the analyses. For the three tit species, most synchrony was across blue tit and great tit, with distinct coefficients for coal tit. The model with shared coefficients for blue tit and great tit was the model with highest posterior probability for all covariates aside from the suburban or rural and ground frost covariates. In the case of the former, the model with blue tit and coal tit shared had the highest posterior mass, while the latter was shared across great tit and coal tit.

The posterior means of the ICCs were close to 1 for blue tit and great tit (0.903, 95% credible interval (0.852, 0.947); and 0.912, 95% credible interval (0.857, 0.957), respectively), suggesting that the majority of unexplained variation for these species was largely synchronous with the other two species in the joint model. The estimate for coal tit, however, was lower at 0.494 (with 95% credible interval [0.411, 0.591]), suggesting that additional asynchronous variation was inherent in the data for this species. This does agree largely with the species that were most frequently shared for the regression coefficients.

In the second analysis, much more of the unexplained variation on average was asynchronous and the magnitude of the random‐effect variances was also greater, probably reflecting the greater tendency of these species to form flocks at feeding sites. This was particularly the case for house sparrow, with posterior mean of 0.205 (0.154, 0.256) for the ICC associated with this species. Greenfinch and chaffinch showed comparatively more synchrony (ICCs of 0.308 (0.211, 0.399) and 0.495 (0.344, 0.648), respectively), but these were both still below the lowest value estimated for the three species of tits. Similarly, greater uncertainty was found across models with regard to which coefficients should be shared, with the preferred pairwise combination only having around 50% posterior support for each of the three time‐invariant covariates. In these three covariates, all three pairwise combinations had greater than 10% posterior support. For each of these coefficients, however, the greenfinch and chaffinch shared parameter had the highest posterior support. For the time‐varying covariates, greater certainty was attributed to a single model, but the shared pair of species in each case was different. For the sparrowhawk covariate, greenfinch and chaffinch shared a parameter value. For collared dove this pair was house sparrow and greenfinch, while for ground frost it was house sparrow and chaffinch.

The results also suggest that the Tweedie variance parameters, namely ϕ and *p*, should have distinct values for all three species in the first analysis, with marginal posterior probabilities of 1 in each case. In the second analysis, the data supported the model with unique values for ϕ (posterior marginal of 1) and *p* shared across house sparrow and chaffinch (posterior marginal of 0.835). The tit species analysis was rerun without model selection on the Tweedie parameters; that is, a single parameter value was assumed across all three species, the results of which can be found in the supporting information.

Density dependence, that is, intraspecific interactions, was found to be highly significant for each of the three tit species (BT: −0.0260 [−0.0298, −0.0221]; GT: −0.0298 [−0.0339, −0.0260]; CT: −0.0333 [−0.0404, −0.0464]). In the grain‐feeding species analysis, no evidence in support of density dependence was found for house sparrow, but there was strong evidence to suggest density‐dependent mechanisms present in greenfinch and chaffinch dynamics (−0.0142 (−0.0195, −0.0093) and −0.0282 (−0.0330, −0.0228), respectively). These results agree well with the analyses conducted in Swallow et al. (2015), where once again no evidence supporting the presence of density‐dependence effects was found in house sparrow. No significant interspecific interactions were found for any combination of the three tit species. In the second analysis, the interspecific interactions were found between some of the species pairs. Both positive and negative interactions were found between different species in the second analysis. The posterior means suggest there is a significant positive effect of greenfinch on house sparrow (0.0327 [0.0258, 0.0401]), while similarly there is a negative effect of chaffinch on house sparrow (−0.0462 [−0.0555, −0.0367]).

## 4 Discussion

The model presented in this study is a highly flexible model that can account for and estimate numerous types of interactions that are inherent in many ecological data sets. The method extends the work of Grosbois et al. (2009) and Lahoz‐Monfort et al. (2011) to allow synchrony across species to be estimated both in their response to environmental covariates fitted as fixed effects in the model and in unexplained variation accounted for through random effects. The use of posterior model probabilities estimated using the reversible jump algorithm ensures that all aspects of synchrony are modeled, and enables more specific conclusions to be drawn as to the nature of the synchrony and to which measured covariates this synchrony relates. This method allows species to be grouped together quantitatively according to how they respond to any covariate under consideration, while estimating distinct coefficients for those species that respond in a significantly different way. Although this is possible through comparison of parameter estimates from single‐species models, our framework is a method that quantitatively discriminates between competing models with different combinations of species grouped together according to their response to covariates. It also takes advantage of the increased precision that sharing parameters affords, with synchrony in response to covariates predominantly relating to species with the highest overlap in credible intervals in the single‐species models. Synchrony to covariates in these analyses relates both to species that show no significant relationship with a given covariate and to those covariates that have a significant but similar manner.

There has been a recent trend in joint modeling approaches to multispecies assemblages. Harris (2015) fitted a joint species distribution model and provided more accurate estimates than were obtained through comparison of results from independent, single‐species models. Single‐species models ignore much of the dependencies and correlations that exist between each of the different species and therefore risk attributing some of the variation spuriously to a covariate that may not be having an overall effect.

The ratio of the unexplained variation can also indicate potential covariates that may be missing from the model. If the unexplained variance is largely synchronous, as is the case in blue and great tits, this suggests that any missing covariates (if there are any) are most likely largely global covariates that have a wide‐ranging effect. In the case of coal tit, where the unexplained variation was largely asynchronous, the indication would be that any missing covariates affect this species alone. As a species, the coal tit has more specific habitat requirements than the other tit species considered. McKenzie, Petty, Toms, and Furness (2007) showed that numbers of coal tits visiting gardens were negatively correlated with a measure of the success of conifer cone production. A covariate measuring the distance to the nearest coniferous forest or the success of the cone crop locally may account for a greater amount of variation than the one fitted here.

The flexibility of the model does not come without computational cost. However, the flexibility can be reduced depending on the nature of the application of interest. In this application, greater flexibility was added to the model through species‐specific dependencies of the Tweedie variance parameters. In the case where a single‐parameter distribution is used, such as in Lahoz‐Monfort et al. (2011), this additional complexity would not be required. In these analyses, posterior support for species‐specific parameters was found and, as such, it seems that the greater computational cost of allowing this flexibility was warranted. Compared to models that did not allow these Tweedie parameters to be species‐specific, the corresponding estimates of the species‐specific random‐effect variances were reduced. This suggests that accounting for these differences at the distribution level allows a greater proportion of the variation between species to be directly accounted for and should therefore lead to improved model performance.

In addition, this framework does not require two separate models to be fitted to detect synchrony in relation to measured covariates, something that is required in the model of Lahoz‐Monfort et al. (2011). Simultaneously, the method also calculates synchrony within any species–covariate combination. The ability to detect how species respond similarly or differently to various exogenous factors can provide important information on possible causes of wide‐ranging, ecosystem‐level changes in populations. It can also account for varying levels of interactions and similarities between species. The need to consider changes in biodiversity at an ecosystem level has been suggested previously (e.g. McCarthy, 2011), and this modeling framework allows such modeling to be conducted. The dynamics underpinning the changes in ecological species are inevitably linked, and failing to take these links into consideration will oversimplify or even incorrectly identify drivers of population change. Lahoz‐Monfort et al. (2011) compare the ICCs for models with and without covariates; however, such an approach does not take into consideration the fact that when removing covariates, some of that additional variation could be absorbed by the intercept or alter the dynamics of the remaining variation that was previously attributed to one of the random effects. The method outlined here prevents this problem by directly accounting for the synchrony to measured covariates in a unified approach.

Although fitting independent models or a joint multispecies model with unique coefficients, followed by postanalysis comparison of credible intervals, could be used as a more simplistic method for detecting synchrony in covariates, the approach outlined here offers a more robust method for comparing similarities between coefficient estimates. The agreement between methods is good, but there were occasions when parameters with distinct credible intervals in the independent models were then shared across multiple species in the joint model (e.g., *p* in the ground‐feeding species analysis).

In both analyses, there tended to be little support for models with no synchrony in response to each covariate—that is, unique coefficients for each of the three species—suggesting that synchrony in response to covariates is a phenomenon that should be taken into consideration in models of this type. Merely using the proportion of unexplained variation as a means for estimating total synchrony will therefore tend to underestimate the total level of synchrony inherent in the data, while also reducing precision in comparison with models with shared coefficients.

The posterior means of the regression coefficients were consistent with those from independent species analyses Swallow et al., unpublished data. Where coefficients were shared, these were usually equal to roughly the average of the two corresponding estimates from the independent analyses. The coefficients that were shared corresponded to either cases where the effect was nonsignificant—that is, where 95% credible intervals included zero—and significant coefficients whose 95% credible intervals did not include zero. In comparing the results from the multispecies analyses with those from independent models, the parameters that were shared almost always corresponded with those whose 95% credible intervals had the largest degree of overlap (Table 13). The biggest exception to this was the suburban/rural covariate in the ground‐feeding species, with the model with the highest posterior model probability relating to the two species having the least overlap in credible intervals in the independent analyses. Secondly, the index parameter *p* was shared between house sparrow and chaffinch, which had quite distinct values in the independent models. All parameters that were shared had a large degree of overlap, although there were some with reasonable overlap that were not shared.

**Table 13:** Proportion of overlap of 95% credible intervals from the independent analyses from Swallow et al. (unpublished data) for each pairwise combination of species. The negative values indicate distinct intervals for each of that pairwise species comparison. Bold values relate to the species pair with the highest posterior model probability for that covariate from the joint model

Species	North	East	Sub/rur	S. hawk	C. dove	Grd. frost	*p*	ϕ
BT/GT	**0.87**	**0.59**	0.82	**−0.02**	**0.62**	0.57	−2.75	−0.69
BT/CT	0.03	0.11	**0.34**	−0.70	0.11	0.46	−1.67	−5.40
GT/CT	0.10	0.05	0.24	−0.31	0.20	**0.53**	−2.23	−4.34
HS/GF	0.28	0.68	0.82	−1.15	**0.22**	−0.73	−1.44	−2.18
HS/CF	−0.18	0.51	0.33	−2.58	0.29	**−1.37**	**−2.32**	−5.96
GF/CF	**0.51**	**0.82**	**0.16**	**−0.11**	0.02	0.58	−4.26	−4.43

Accounting for the different shape distributions for each species—that is, allowing *p* and ϕ to be species‐invariant—also reduced the magnitude of asynchronous variance compared to models with these parameters constrained to be equal, particularly for coal tit whose posterior means for these two parameters were most different from the others. The posterior mean of ICC${}_{\mathrm{ct}}$ decreased from 0.494 to 0.414 when the Tweedie parameters remained shared across all species, a bigger change than the other two species suggesting more asynchronous variance in the shared parameter model. As these parameters determine the variance of the Tweedie distribution, allowing them to differ for each species will directly account for differences in variance for each species and, as such, a smaller amount of asynchronous variation would then be expected in the means for each species.

In some cases, it appears that the model is unable to differentiate between inter‐ and intraspecific interactions. Once the interaction with conspecifics has been accounted for, that is, density dependence, there appears to be little additional variation left that can be explained by the number of other species observed at that site. The two species groups considered in the two models were chosen largely because they have similar ecological requirements, and hence, we would expect these interaction covariates to be highly correlated. It seems positive, though, that the model is predominately selecting the number of conspecifics over the number of the other species in the model as the best predictor of variation. No species showed significant density dependence in addition to an effect of another species on it. That is, the changes observed in species counts were affected by either the presence of conspecifics *or* that of at most one other species. This may highlight a difficulty of separating out some of the high‐level interactions between species that are inherent in these multispecies models. However, modeling the simpler ones clearly adds to the understanding of the ecology, rather than ignoring the effects of other birds visiting the feeders at the same time.

The results presented in this analysis suggest that there is indeed a large degree of synchrony in many of the species studied. The drivers of numbers of blue tit and great tit visiting garden feeding stations appear to be particularly strongly correlated, observed through both their tendency for shared covariate parameters and their ICC values. The shared northing and easting parameters suggest similar spatial trends for the two species, which is supported by information presented within Bird Atlas 2007–11 (Balmer et al., 2013, pp. 496–499). Coal tits have shown negative trends in the southeast of the UK (Balmer et al., 2013, pp. 502–503), which is reflected in their unique parameter values. The effect of sparrowhawks and collared doves on the three species seems to be negligible in blue and great tits, but with a small but significant positive association with coal tits. The latter could represent confounding factors that led to sparrowhawks and collared doves recolonizing sites that were also more attractive to coal tits, such as a preference for larger gardens, rather than a causal relationship. All three species have a tendency to make use of garden feeders more in conditions of cold weather Chamberlain et al. (2005), when natural food sources can be harder to access. The positive effect of ground frost on numbers of observed birds suggests a behavioral response of birds entering gardens to access food sources that will be independent of the weather. The strongest effect of this covariate was found in blue tits, with a smaller and equivalent effect on the other two species.

In the case of the ground‐feeding species, our results have shown that there are frequently similar effects of environmental factors on the numbers visiting garden feeding stations. However, in this case the differences between ICCs were much smaller than in the other species group. Greenfinch and chaffinch unsurprisingly were the two species showing most synchrony in their response to environmental covariates. The exceptions to this were in their relationship with collared doves and ground frost. In the former case, numbers of chaffinches were more positively associated with collared doves than the other two species, while greenfinches were less affected by ground frost. Chaffinch and collared dove are more strongly ground‐feeding than greenfinch and frost would be expected to effect ground feeders more than those species using perched feeders. These individual differences in responses to covariates would have been missed if covariate synchrony had not been added to the modeling process.

Multispecies models that account for the complex interactions within and between species have the potential to offer a much greater understanding of the underlying dynamics to which species are responding, either individually or at an ecosystem level. Although the subtleties of the complex processes will always be simplified to some degree when using mathematical models, the method outlined here allows some of those complexities to be accounted for directly. Joint species responses to specific covariates may suggest areas for further research or indicate areas for management that can benefit a whole ecosystem rather than just its constituent parts. Long‐term studies, such as the GBFS, provide invaluable insight into a community of species that are subject to the same environmental factors; the methods used to analyze such data should really take this into consideration. Given the increasing pressures on land, and a growing degree of urbanization, there is a clear need to understand the ecological processes driving the changes that we are seeing within wild bird populations. New statistical approaches, such as this, provide an opportunity to look at these processes across species in a more effective manner.

## Conflict of Interest

None declared.
