# Supplementary material for: Identifying multispecies synchrony in response to environmental covariates
Source: Ecol Evol. 2016 Nov 4;6(23):8515–25. doi: 10.1002/ece3.2518 (PMC5167035; doi:10.1002/ece3.2518)
Supplement: Supplementary file 1 [file ECE3-6-8515-s001.pdf]

Running title: Multi-species synchrony to covariates
Number of words:
Number of tables: 0
Number of figures: 0
Number of references: 0

Identifying multi-species synchrony in response to environmental covariates (Appendices).

Ben Swallow *[1,2] Ruth King [1,3]

Stephen T. Buckland [1] and Mike P. Toms[4]

1. *Centre for Research into Ecological and Environmental Modelling, School of Mathematics and Statistics, University of St Andrews, KY16 9LZ, UK*
2. *Atmospheric Chemistry Research Group, University of Bristol, Bristol BS8 1TS*
3. *School of Mathetmatics and Statistics, University of Edinburgh, Edinburgh, EH9 3FD*
4. *British Trust for Ornithology, The Nunnery, Thetford, Norfolk, IP24 2PU, UK*

*Corresponding author. Current address:
Ben Swallow
Atmospheric Chemistry Group, University of Bristol, Bristol BS8 1TS
ben.swallow@bristol.ac.uk

Date of submission: 10th February 2016

1 Appendix 1

1.1 Detecting synchrony to measured covariates

Below we outline the RJMCMC algorithm for detecting synchrony in response to measured covariates.

STEP 1: Update the parameters

i) Assume that at iteration t we are in model m, with model parameters $\boldsymbol{\theta}_m$. We update parameters $\boldsymbol{\theta}_m$ conditional on model m using a single update Metropolis-Hastings algorithm.

STEP 2: Update the model

i) Conditional on the parameter values, we propose to move to an alternative model denoted m'.
Initially, we select one of the regression covariate parameters or Tweedie variance parameters with equal probability, denoted $\theta_{j,m}$. For the remainder of this iteration we concern ourselves with this parameter only.

ii) We then choose with probability $\frac{1}{2}$, whether we are going to split or merge groups of species.

(a) If we choose to split an existing group of species, moving from model m to m', where model m' has one greater number of unique values for one of the model parameters that can be shared:

Assume parameter $\theta_{j,m}$ currently has n^* unique values, where $1 \leq n^* \leq n_s$ and n_s is the total number of species under consideration. One of these subsets of species with the same parameter value, denoted $\{\boldsymbol{S}_h\}$, is selected with equal probability from the n^* possible subsets. Let $\theta_{j,m}\{\boldsymbol{S}_h\}$ be of length $|\boldsymbol{S}_h|$. Then if $|\boldsymbol{S}_h| = 1$, we automatically reject the move and move to iteration $t+1$, with the model remaining model m.

If $|\boldsymbol{S}_h| > 1$, we split $\boldsymbol{S}_h$ into two mutually exclusive subsets denoted $\boldsymbol{S}_{h1}$ and $\boldsymbol{S}_{h2}$, with $2^{|\boldsymbol{S}_h|-1}-1$ possible combinations. We simulate $u \sim q(.)$ and set $\theta_{j,m}\{\boldsymbol{S}_{h1}\}' = \theta_{j,m}\{\boldsymbol{S}_h\}+u$ and $\theta_{j,m}\{\boldsymbol{S}_{h2}\}' = \theta_{j,m}\{\boldsymbol{S}_h\}-u$. Thus, model m' has n^*+1 unique values from parameter $\theta_{j,m'}$. The acceptance probability is then $\min(1, A)$, where:

$$A = \frac{\pi(\boldsymbol{\theta}'_m, m'|\boldsymbol{y})P(m|m')\,|J|}{\pi(\boldsymbol{\theta}_m, m|\boldsymbol{y})P(m'|m)q(u)}$$

and $|J| = 2$, $P(m'|m) = 1/n^* \times 1/\left(2^{|\boldsymbol{S}_h|-1}-1\right)$ and $P(m|m') = \binom{n^*+1}{2}^{-1}$.

(b) If we choose to merge two existing groups of species, we propose to move from model m' to model m:

Suppose we are currently in model m' and there are currently n^*+1 groups with unique values for this parameter. If there are no groups to merge, we automatically reject this move and move to iteration $t+1$.

Else, we randomly choose two of these groups denoted $\boldsymbol{S}'_{h_1}$ and $\boldsymbol{S}'_{h_2}$. We set $\boldsymbol{\theta}_{j,m}\{\boldsymbol{S}_h\} = \left(\boldsymbol{\theta}_{j,m'}\{\boldsymbol{S}_{h_1}\}' + \boldsymbol{\theta}_{j,m'}\{\boldsymbol{S}_{h_2}\}'\right)/2$ and $u = \frac{1}{2}\left(\boldsymbol{\theta}_{j,m'}\{\boldsymbol{S}_{h_1}\}' - \boldsymbol{\theta}_{j,m'}\{\boldsymbol{S}_{h_2}\}'\right)$.

The acceptance probability is then equal to the reciprocal of A defined above.

iii) Increase the iteration number by one and repeat the first four steps until the required number of iterations has been achieved.

2 Appendix 2

Here we include results from the analysis of the three tit species where the parameters ϕ and p are assumed constant across all three species for comparison. The greatest differences observed were in the asynchronous coal tit random effect variance, and hence the ICC_{ct} coefficient. There was also some changes in the posterior model probabilities associated with the northing and suburban/rural coefficients.

Table 1 Blue tit, great tit and coal tit multi-species model. Intercept and density-dependence parameters are species-specific, with the reversible jump algorithm used to test for synchrony across the three species for all other regression covariate parameters. Posterior means and 95% symmetric credible intervals are presented.

Parameter	Covariate	Posterior mean	95%CI
$\alpha\{bt\}$	Intercept	-0.0343	(-0.0410,-0.0269)
$\alpha\{gt\}$	Intercept	-0.0273	(-0.0335,-0.0202)
$\alpha\{ct\}$	Intercept	-0.0419	(-0.0524,-0.0320)
$\beta_1\{bt\}$	Northing	-0.0075	(-0.0142,-0.0003)
$\beta_1\{gt\}$	Northing	-0.0078	(-0.0140,-0.0009)
$\beta_1\{ct\}$	Northing	0.0022	(-0.0122,0.0207)
$\beta_2\{bt\}$	Easting	-0.0096	(-0.0161,-0.0028)
$\beta_2\{gt\}$	Easting	-0.0096	(-0.0161,-0.0028)
$\beta_2\{ct\}$	Easting	-0.0358	(-0.0506,-0.0220)
$\beta_3\{bt\}$	Sub/rur	-0.0160	(-0.0227,-0.0090)
$\beta_3\{gt\}$	Sub/rur	-0.0158	(-0.0229,-0.0079)
$\beta_3\{ct\}$	Sub/rur	-0.0128	(-0.0215,-0.0011)
$\nu_{bt,bt}$	Dens dep	-0.0249	(-0.0282,-0.0213)
$\nu_{gt,gt}$	Dens dep	-0.0287	(-0.0325,-0.0249)
$\nu_{ct,ct}$	Dens dep	-0.0443	(-0.0493,-0.0368)
$\gamma_1\{bt\}$	Sparrowhawk	-0.0020	(-0.0067,0.0025)
$\gamma_1\{gt\}$	Sparrowhawk	-0.0015	(-0.0066,0.0035)
$\gamma_1\{ct\}$	Sparrowhawk	0.0209	(0.0131,0.0283)
$\gamma_2\{bt\}$	Collared dove	-0.0022	(-0.0054,0.0014)
$\gamma_2\{gt\}$	Collared dove	-0.0022	(-0.0054,0.0014)
$\gamma_2\{ct\}$	Collared dove	0.0136	(0.0057,0.0211)
$\gamma_3\{bt\}$	Ground frost	0.0167	(0.0120,0.0221)
$\gamma_3\{gt\}$	Ground frost	0.0138	(0.0091,0.0192)
$\gamma_3\{ct\}$	Ground frost	0.0140	(0.0091,0.0197)
ϕ	-	0.2437	(0.2385,0.2489)
p	-	1.2112	(1.2044,1.2183)

Table 2 Blue tit, great tit and coal tit multi-species model. Posterior means and 95% symmetric credible intervals for the random effect variances and synchrony coefficeints.

Parameter	Posterior mean	95%CI
σ^2_{ϵ}	0.0046	(0.0040,0.0053)
σ^2_{bt}	0.0004	(0.0002,0.0007)
σ^2_{gt}	0.0003	(0.0002,0.0005)
σ^2_{ct}	0.0066	(0.0051,0.0085)
ICC_{bt}	0.919	(0.873,0.937)
ICC_{gt}	0.935	(0.895,0.950)
ICC_{ct}	0.414	(0.341,0.445)

Table 3 Marginal posterior probabilities for the sharing of the time-invariant covariate parameters $\boldsymbol{\beta}$ from the model in Table 2, corresponding to the time-invariant covariates. $\{bt, gt\}$, for example, corresponds to the model where the coefficient is shared across blue tit and great tit.

Northing		Easting		Sub/rur	
Model	MPP	Model	MPP	Model	MPP
$\{bt, gt\}, \{ct\}$	0.493	$\{bt, gt\}, \{ct\}$	0.988	$\{gt, ct\}, \{bt\}$	0.566
$\{gt, ct\}, \{bt\}$	0.397	$\{bt\}\{gt\}, \{ct\}$	0.012	$\{bt, gt\}, \{ct\}$	0.388
$\{bt, ct\}, \{gt\}$	0.103			$\{bt, ct\}, \{gt\}$	0.044
$\{bt\}, \{gt\}, \{ct\}$	0.007			$\{bt\}, \{gt\}, \{ct\}$	0.003

Table 4 Marginal posterior probabilities for from the model in Table 2, corresponding to the time-varying covariates $\boldsymbol{\gamma}$. $\{bt, gt\}$, for example, corresponds to the model where the coefficient is shared across blue tit and great tit.

Sparrowhawk		Collared Dove		Ground frost	
Model	MPP	Model	MPP	Model	MPP
$\{bt, gt\}, \{ct\}$	0.892	$\{bt, gt\}, \{ct\}$	0.989	$\{gt, ct\}, \{bt\}$	0.902
$\{bt\}, \{gt\}, \{ct\}$	0.108	$\{bt\}, \{gt\}, \{ct\}$	0.011	$\{bt, ct\}, \{gt\}$	0.097
				$\{bt\}, \{gt\}, \{ct\}$	0.001
